# The impact of an early_exposure program on medical students’ interest in and knowledge of rural medical practices: a questionnaire survey

**DOI:** 10.1186/s12930-015-0021-8

**Published:** 2015-04-14

**Authors:** Naoto Ishimaru, Ayumi Takayashiki, Takami Maeno, Yurika Kawamura, Hiroshi Kurihara, Tetsuhiro Maeno

**Affiliations:** Department of Internal Medicine, Akashi Medical Center, Ohkubo-cho Yagi, Akashi, Hyogo 674-0063 Japan; Department of Primary Care and Medical Education, University of Tsukuba, 1-1-1 Tennodai, Tsukuba, Ibaraki 305-8575 Japan; Community-Based Medicine Training Station, Tsukuba University Hospital, 2-1-1 Amakubo, Tsukuba, Ibaraki 305-8576 Japan

**Keywords:** Rural practice, Early exposure, Undergraduate education

## Abstract

**Background:**

Many medical students in Japan were brought up in urban areas, thus rural medical practice is often unfamiliar to them. The University of Tsukuba created a one-day early_exposure program to provide freshman students with experience in rural practices. This study was designed to clarify how this one-day early_exposure program affected medical students’ attitudes toward and knowledge of rural practices.

**Findings:**

First-year medical students (n = 103) were assigned to one of seven rural clinics in which they experienced rural practice for one day. A pre- and post-program questionnaire, rated on a 5-point Likert scale, was administered to assess students’ interest in and knowledge of rural medical practice, with higher scores indicating greater interest and knowledge. Respondents who gave answers of 4 or 5 were defined as having high interest and knowledge. One hundred and one (98.1%) responses were received from students. After the program, the percentage of students interested in rural medical practices was increased (pre- and post-program: 39.0% and 61.0%, respectively; *P* < .001), as was the number of students who wanted to become physicians in a rural medical practice (pre- and post-program: 53.0% and 73.0%, respectively; *P* < .01).

**Conclusions:**

Our one-day early_exposure program demonstrated a positive impact on medical students’ interest in and knowledge of rural medical practice. Further follow-up surveys are needed to clarify whether these effects are sustained long-term.

## Introduction

The shortage of physicians in rural areas is a longstanding and serious problem in Japan and worldwide [1,2]. National policymakers and educators continue their attempts to overcome the challenge of retaining a physician workforce in rural areas [3]. In this regard, community-based education (CBE) has been reported to have some positive effect on recruitment of rural physicians [4,5].

It has been suggested that medical students brought up in rural areas have a preference for rural medical practices [6] and are more likely to return to rural areas after training [7]. However, an observational study reported that many medical school students in Japan were raised in urban areas, while only 3.3% of medical students were raised in rural areas [8].

Thus, a possible factor contributing to a one-sided physician distribution is the lack of familiarity with rural experience. Recent reports suggested that exposing medical students of urban backgrounds to rural medical practices increases their interest in opportunities for such placement [9,10].

**Figure 1:**
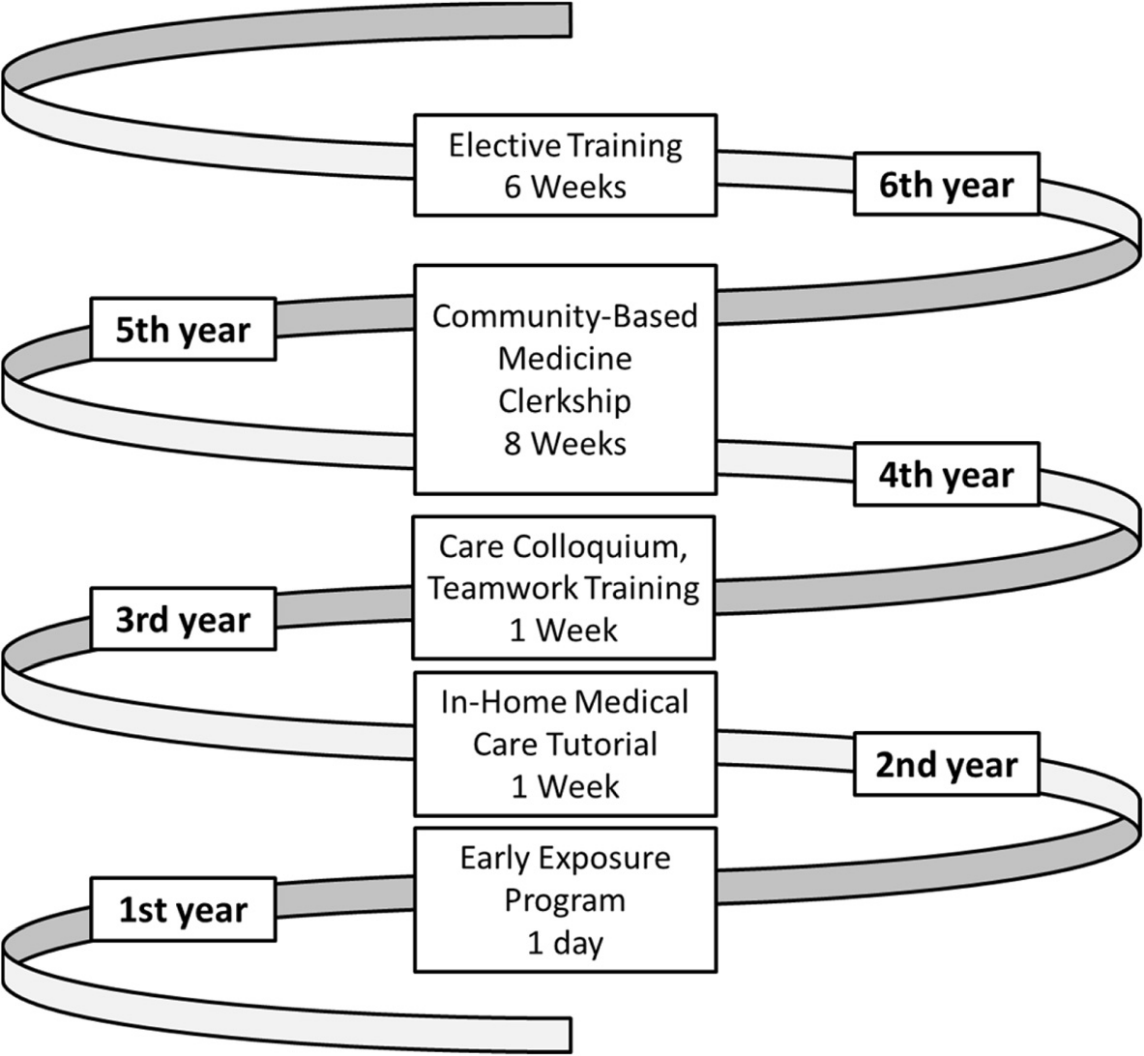
Community-based learning curriculum. In their first year, all students are required to take part in a one-day early_exposure program in order to become familiar with rural practices. In their second year, care conferences are held and care plans are designed according to a home-care scenario as part of a one-week course called the “In-home Medical Care Tutorial”. In their third year, the medical students discuss team-based care in collaboration with nursing, pharmacy, and clinical laboratory technician students in a one-week-long course called “Teamwork Training”. On-the-job training in a rural medical practice is also undertaken in each student’s fifth year during an eight-week course called the ‘Community-Based Medicine Clerkship’. In their sixth year, a one- to six-week optional rural practice training is also available as an elective.

As a part of CBE, early_exposure programs for rural areas have been introduced by many medical schools, including those in Japan [11]. The University of Tsukuba, situated in a rural area with a severe shortage of physicians (1.5 practicing physicians per 1000 residents, well below the Organisation for Economic Co-operation and Development average of 3.1), recently created a CBE program (Figure 1). The curriculum is based on the concept of spiral learning [12]. In their first year, every student is required to take part in a one-day early_exposure program to enable them to directly experience rural practice.

Evidence has suggested that early experience in rural exposure programs can have a positive influence on students’ preferences for and attitudes toward working in rural medical practices [13,14], as well as their perceptions of rural primary care [15]. However, the duration of these exposure programs is generally over 75 hours, and the impact of a one-day exposure program on freshman students’ interest in rural practice remains uncertain [16].

The aim of this study was to clarify the effects of a one-day early_exposure program on medical students’ interest in and knowledge of rural medical practices in terms of the following factors: interest in rural medical practice and understanding the rural physician’s role.

## Methods

### Design and subjects

All first-year medical students at the University of Tsukuba in 2010 were asked to complete a self-administered questionnaire both during their classroom orientation before the early_exposure program, and immediately following the program but while still at the rural practice.

Students’ gender, attitudes toward and knowledge of rural practices, and preferred future work location were recorded in the questionnaire. Their preferences for each of the following work locations were recorded: large city, major urban area, town or village, and rural area. These preferences were rated on a 5-point Likert scale ranging from 1 (“I do not prefer”) to 5 (“I prefer very much”). Responses of either 4 or 5 were considered to demonstrate a preference for that work location; the number of students who preferred rural areas was thus calculated. In addition, their interests in and knowledge of rural practice were assessed with the following items, respectively: “Are you interested in rural practice?” and “Do you understand the physicians’ roles in rural practice?” These items were rated on a 5-point Likert scale ranging from 1 (“I have no interest” and “I do not understand”, respectively) to 5 (“I am very interested” and “I understand very well”, respectively). Responses of either 4 or 5 were considered to indicate high interest in and knowledge of rural medical practices.

### The early_exposure program

First-year students at the University of Tsukuba are assigned to one of seven rural clinics throughout Ibaraki in four student units, where they can experience rural practice for one day. Each unit is supervised by a faculty physician.

### Statistical analyses

McNemar’s test was used to evaluate pre- and post-program differences in work location preferences and the proportions of respondents with high interest and knowledge regarding rural practice. The Chi-square test was used to analyse differences in basic work location preferences and the relationship between gender and post-program interest in and knowledge of rural medical practices. Statistical significance was set at *P* < .05.

### Ethical considerations

The original purpose of this questionnaire survey was to evaluate an educational program for its improvement. The data from the pre- and post-program evaluations were used after removing all of the students’ personal information.

When given the questionnaire, participants were informed that no personally identifiable information would be used in the results, that there was no penalty for non-participation, and that the questionnaire had no bearing on their grade.

## Findings

Of the 103 students, responses were received from 101 (98.1%); 33 (32.7%) were women. A preference to work in a large city or major urban area in the future was reported by the majority of students pre-program (64; 63.4%), while the students’ preferences were lower for working in a town or village (34; 33.7%) or rural area (17; 16.8%; Table 1). The distribution of students who preferred a rural area, as stratified by their other basic work location preferences, is shown in Table 1. For example, among the 64 students who preferred to work in a large city, a rural area was also preferred by only 8 (12.5%).

**Table 1:** **Students’ preference for rural area by basic work location preference (N = 101)**

**Work location preference**	**Subjects n (%)**	**Students preferring rural practice n (%)**
Large city	64 (63.4)	8/64 (12.5)
Major urban area	64 (63.4)	12/64 (18.8)
Town or village	34 (33.7)	12/34 (35.3)
Rural area	17 (16.8)	―

All data are expressed as numbers with percentages in parentheses.

**Figure 2:**
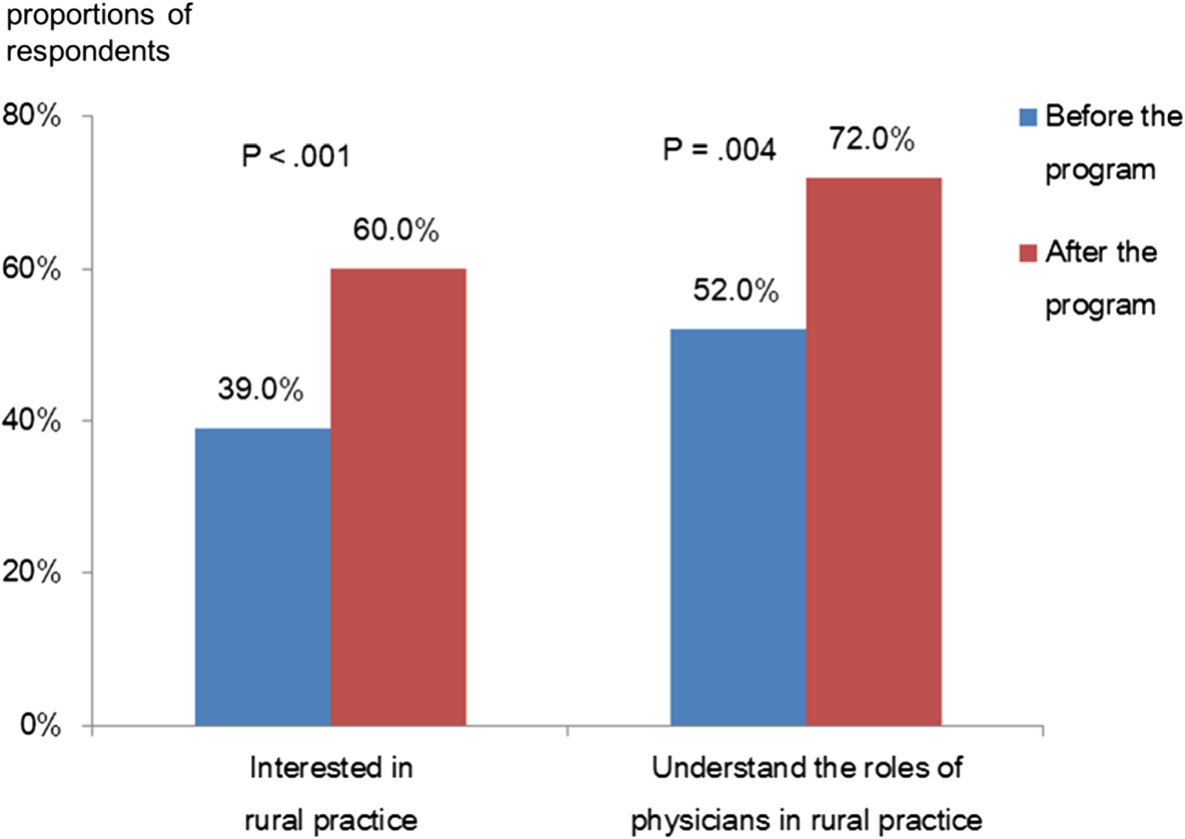
Students’ changes in interest and knowledge pre- and post-program (n = 101). All data are expressed as percentages of students both pre- and post-program who reported high interest in rural practice and good understanding of physicians’ roles in rural practice. Responses to these items of either 4 or 5 on a Likert scale were considered to indicate high interest in and knowledge of rural medical practice. McNemar’s test was used to examine the pre- and post-program differences in proportions of respondents with high interest and knowledge.

The numbers of students who reported high interest in rural practice and good understanding of physicians’ roles in rural practice pre- and post-program are shown in Figure 2. After the program, there was a significant increase in the number of students interested in rural practice (pre- and post-program: 39.0% and 60.0%, respectively). Similarly, there was a significant increase in the number of students who reported a good understanding of the roles of physicians in rural practices (pre- and post-program: 52.0% and 72.0%, respectively).

These results and our comparison of the students’ pre- and post-program interest and understanding of rural medical practices by work location preference are shown in Table 2. The results indicated that even among students who preferred large cities, there was an increase after the program both in the number of students interested in rural practices (pre- and post-program: 26.6% and 54.7%, respectively) and in those who had a good understanding of physicians’ roles in rural practice (pre- and post-program: 46.9% and 73.4%, respectively). Furthermore, pre-program data showed that students who preferred towns or villages as well as rural areas reported both a high initial interest in rural practice (towns or villages and rural areas: 73.5% and 88.2%, respectively) and a good understanding of physicians’ roles in rural practice (towns or villages and rural areas: 64.7% and 64.7%, respectively). Student gender was not associated with either post-program interest in or knowledge of rural medical practice (*P* = 0.89 and *P* = 0.38, respectively).

**Table 2:** **Pre- and post-program changes in students’ interest in and knowledge of rural practice by basic work location preference**

		**Interested in rural practice**			**Understand physicians’ roles in rural practice**		
**Work location preference**	**Subjects**	**Pre-program**	**Post-program**	***P*** **-value**	**Pre-program**	**Post-program**	***P*** **-value**
	**n**	**n (%)**	**n (%)**		**n (%)**	**n (%)**	
Large city	64	17 (26.6)	35 (54.7)	< .001	30 (46.9)	47 (73.4)	.003
Major urban area	64	29 (45.3)	42 (65.6)	.002	40 (62.5)	46 (71.9)	.29
Town or village	34	25 (73.5)	29 (85.3)	.22	22 (64.7)	27 (79.4)	.23
Rural area	17	15 (88.2)	16 (94.1)	1.00	11 (64.7)	13 (76.5)	.69

All data are expressed as numbers and percentages in parentheses.

McNemar’s test was used to examine pre- and post-program differences in the proportions of respondents with high interest and knowledge.

## Discussion

The impact of early_exposure programs on freshman students’ interest in rural practices and their understanding of physicians’ roles in these practices was underscored by these findings. The one-day early_exposure program also had a positive impact on their attitudes and knowledge towards rural practice.

Several Asia Pacific countries have now developed CBE programs in rural areas [17,18] that are offered as alternative curricular options for fourth-year and fifth-year medical students. Although our program also contains a community-based medicine clerkship in the fifth year, the unique aspect of this early_exposure program is that all medical students participate in the first semester of their first year. A previous study showed that early intentions at the start of a student’s medical training were associated with the expressed intention to pursue a rural placement [19], suggesting the importance of launching the early_exposure program just after medical course admission.

Another important finding was that the positive impact on interest in and knowledge of rural practices was seen even among the students who preferred working in large cities. A preference for future work options in both large cities and rural areas was reported by only 12.5% of students before the program; thus, this program could effectively influence such students towards considering rural practices, which could in turn improve the geographic distribution of physicians.

The current study has several limitations. First, the impact of this early_exposure program remains at Kirkpatrick level 1 [20], which corresponds to the participants’ reaction after the program. There is no confirmatory evidence that an early_exposure program produces any sustainable change in attitude or lasting change in knowledge of rural practice. We conducted an eight-week community-based medicine clerkship for fifth-year students, and we wish to undertake further research to determine whether the current impact on interest in and knowledge of rural practices can be sustained. Second, we describe a program that was implemented in only one medical school. However, the institution surveyed was a local university that was applying a comprehensive community-based learning curriculum. Thus, the benefit of our findings may be applicable to other short-term, compulsory, early_exposure programs in local universities.

## Conclusion

The one-day early_exposure program was found to be a valuable enrichment experience for new students. Further follow-up surveys are essential to clarify whether its impact can be sustained.
